# Dielectrophoresis Aligned Single-Walled Carbon Nanotubes as pH Sensors

**DOI:** 10.3390/bios1010023

**Published:** 2011-01-31

**Authors:** Pengfei Li, Caleb M. Martin, Kan Kan Yeung, Wei Xue

**Affiliations:** Mechanical Engineering, School of Engineering and Computer Science, Washington State University, 14204 NE Salmon Creek Avenue, Vancouver, WA 98686, USA; E-Mails: pengfei_li@wsu.edu (P.L.); cmartin25@wsu.edu (C.M.M.); kandy@wsu.edu (K.K.Y.)

**Keywords:** single-walled carbon nanotube (SWNT), dielectrophoresis, alignment, pH sensor, response time

## Abstract

Here we report the fabrication and characterization of pH sensors using aligned single-walled carbon nanotubes (SWNTs). The SWNTs are dispersed in deionized (DI) water after chemical functionalization and filtration. They are deposited and organized on silicon substrates with the dielectrophoresis process. Electrodes with “teeth”-like patterns—fabricated with photolithography and wet etching—are used to generate concentrated electric fields and strong dielectrophoretic forces for the SWNTs to deposit and align in desired locations. The device fabrication is inexpensive, solution-based, and conducted at room temperature. The devices are used as pH sensors with the electrodes as the testing pads and the dielectrophoretically captured SWNTs as the sensing elements. When exposed to aqueous solutions with various pH values, the SWNTs change their resistance accordingly. The SWNT-based sensors demonstrate a linear relationship between the sensor resistance and the pH values in the range of 5–9. The characterization of multiple sensors proves that their pH sensitivity is highly repeatable. The real-time data acquisition shows that the sensor response time depends on the pH value, ranging from 2.26 s for the pH-5 solution to 23.82 s for the pH-9 solution. The long-term stability tests illustrate that the sensors can maintain their original sensitivity for a long period of time. The simple fabrication process, high sensitivity, and fast response of the SWNT-based sensors facilitate their applications in a wide range of areas.

## 1. Introduction

Since its discovery, the single-walled carbon nanotube (SWNT) has been intensively studied by researchers due to its special electrical and physical characteristics. According to many recent publications, there are two important directions for SWNT research in engineering. One direction explores how to manipulate SWNTs effectively and efficiently using different approaches, given that SWNTs are extremely small and hard to be placed in desired locations. The other direction investigates how to apply SWNTs in different areas and develop practical devices. Among various applications, sensor research has been one of the most widely studied topics for SWNT-based devices. An immense variety of sensors have been developed such as flow sensors [1,2], strain sensors [3,4], chemical sensors [5], gas sensors [6,7,8,9] and pH sensors [10,11,12,13]. The inherent properties of SWNTs such as small dimensions and high conductivity make them promising candidates for sensing applications. The SWNT-based sensors have exhibited excellent performance due to the considerable number of sites on the tubular surfaces where molecules can react. These sensors are usually minuscule in size and therefore have great potential to be integrated with electronic devices or circuits for signal processing. For example, bioelectronic chips based on SWNTs and field-effect transistors (FETs) have been developed by a number of groups; they have demonstrated high performance as biosensors [14,15,16].

Many of these sensors rely on the accurate and real-time detection of pH value which is one of the most critical measurable parameters in many fields such as medicine, chemistry, biology, material science, *etc*. The pH value is a quantity commonly used to monitor the properties of solutions. However, most commercial pH sensors on the market are expensive, relatively large, and incompatible with integrated circuits. By contrast, SWNT-based pH sensors are not only affordable but also small in size. Furthermore, SWNTs can be easily integrated into electronic devices as the sensing elements. While the SWNT-based sensors have demonstrated high pH sensitivity [17,18], the fabrication processes of these sensors are often complex and difficult to be extended to a large scale. Dielectrophoresis, a simple but versatile method, has proven to be effective in organizing single-walled carbon nanotubes (SWNTs) in small and large scales, resulting in devices with predictable properties and performance [19,20]. This method can be conducted at room temperature with low voltages. In addition, a number of parameters such as solution concentration, deposition time, AC source amplitude, and frequency can be adjusted to optimize the quality of the aligned SWNTs. More importantly, dielectrophoresis can be easily incorporated into device fabrication [21,22] and has the potential to be used in wafer-level deposition for mass production of SWNT-based sensors [23].

In this research, we implement dielectrophoresis into pH sensor fabrication. This is based on our previous investigation on the alignment of SWNT thin films and individual SWNTs [24]. Pristine SWNTs are functionalized with a mixture of sulfuric acid and nitric acid to increase their solubility in de-ionized (DI) water. Au electrodes on silicon substrates are prepared with photolithography and wet etching. SWNT bundles are deposited over the “teeth”-like electrodes with dielectrophoresis; they are aligned to follow the generated electric field in the process. The devices are used as pH sensors with the electrodes as the testing pads and the aligned SWNTs as the sensing elements. The deposited SWNTs are inspected with a scanning electron microscope (SEM) to study the quality of the alignment and the influence of solutions on them. The SEM characterization shows that the bonding between the SWNTs and the electrodes is affected by the liquid placing and removing steps in the beginning of the pH sensing procedure. The aligned SWNTs demonstrate high sensitivity towards pH solutions. When pH value is reduced, the resistance of the sensor decreases, and vice versa. The sensing repeatability across multiple sensors, the response time, and the stability of the sensor over time are described and discussed in this paper.

## 2. Materials and Experiments

**Figure 1:**
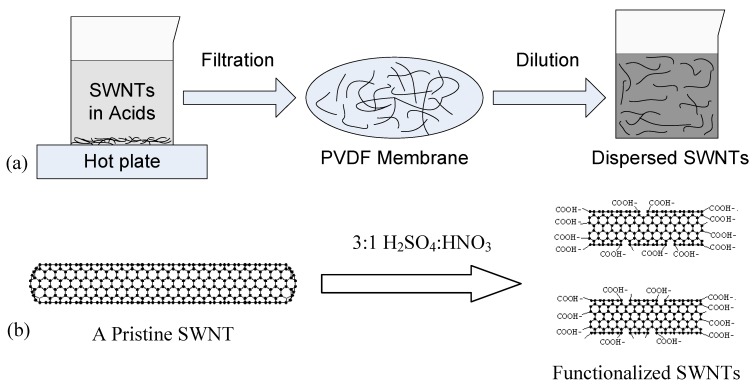
**(a)** Surface functionalization process of SWNTs with acid treatment, filtration and dilution. **(b)** Scheme of the chemical functionalization of a SWNT.

The pristine SWNTs (outer diameter: <2 nm, length: 5–15 μm, purity: >90%), in the form of powder, are purchased from SES Research (Houston, TX). Because dielectrophoresis is a solution-based method, it requires that the deposited material is able to move and rotate freely in a medium. Therefore, the pristine SWNTs are treated with a three-step process to increase their solubility in DI water, as shown in Figure 1(a). First, the SWNTs are treated in a mixture of acids H_2_SO_4_:HNO_3_ (volume ratio 3:1) at 110 °C for 45 min to introduce carboxyl groups (–COOH) on the surfaces and open ends (Figure 1(b)) [25]. Second, the functionalized SWNTs are diluted in DI water and filtered with a polyvinylidene fluoride (PVDF) filtration membrane (with an average pore diameter of 0.22 μm) repeatedly for 5–6 times until the pH value of the dispersion reaches five. Third, a 30-min ultrasonic process is performed to uniformly disperse the SWNTs in DI water. The concentration of the SWNTs in the final solution is approximately 0.1 mg/mL.

**Figure 2:**
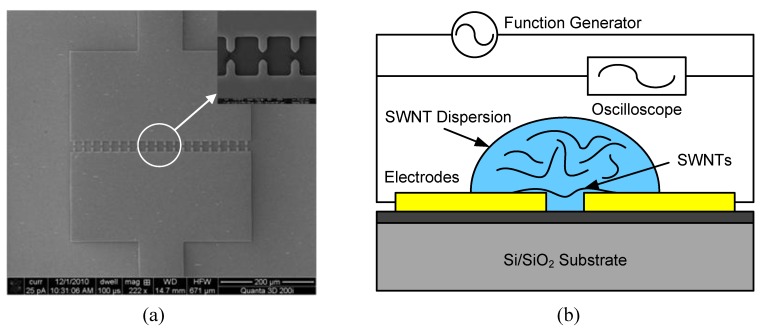
**(a)** SEM images of the “teeth”-like electrode design. The enlarged image shows the electrodes. **(b)** Experimental setup for the dielectrophoresis of SWNTs.

The electrodes are fabricated on 4-inch silicon wafers. The wafers are covered with a 200-nm-thick thermal-grown SiO_2_ insulating layer. Metal layers of Cr (100 nm, adhesion material) and Au (200 nm, electrode material) are coated on the wafer surface with sputtering. The electrodes are designed as “teeth”-like structures which can enable a highly concentrated electric field in between. SEM images of the entire device and the enlarged electrode area are illustrated in Figure 2(a). The width of each “tooth” is 6 μm; the gap between two opposite “teeth” is 3 μm. The schematic diagram of the dielectrophoresis process is shown in Figure 2(b). A function generator (Agilent Technologies 81150A) serves as the AC source to generate electric field between the electrodes and an oscilloscope (Agilent Technologies MSO 7054A) monitors the voltage change in real time during the dielectrophoresis process. The frequency and the peak-to-peak voltage of the AC signal are 5 MHz and 10 V, respectively. When the SWNTs in DI water are exposed to the electric field, the induced dielectrophoretic force can move and rotate them along the electric field lines. Eventually, the SWNTs are deposited on the substrate to connect the electrodes. The van der Waals force strengthens the bonding between the aligned SWNTs and the electrodes.

**Figure 3:**
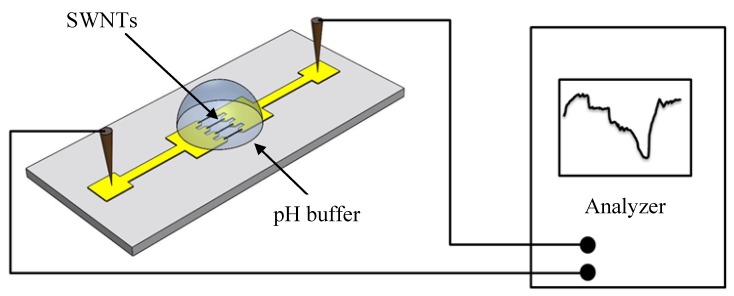
Schematic diagram of the pH sensing circuit.

With the dielectrophoresis aligned SWNTs, the device is used as a pH sensor with the electrodes as the testing pads and the SWNTs as the sensing elements. The experimental setup to characterize the pH sensitivity of the sensor is shown in Figure 3. The pH buffer solutions, purchased from Fisher Scientific (Fair Lawn, NJ), are carefully placed on the sensor using syringes to cover the aligned SWNTs. The sensor is exposed to five different buffer solutions, with pH values ranging from 5 to 9, in a controlled sequence. To measure the effects of pH solutions on the SWNTs, the sensor is directly connected to a semiconductor device analyzer (Agilent Technologies B1500A) with two probes sticking onto the electrode pads. The semiconductor device analyzer is able to supply a controllable DC current and monitor the voltage variation at the same time. Therefore, the real-time resistance of the sensor can be recorded. The sampling rate of the data acquisition is set as 1 point per sec. The DC current source is fixed as 50 µA. Each pH solution measurement takes approximate 50 s, during which the signal of the sensor becomes stabilized.

## 3. Results and Discussion

### 3.1. Dielectrophoresis

During the dielectrophoresis process, the AC source applied across the electrodes generates a non-uniform electric field, which causes the electrons and protons in the SWNTs to move away from their balanced positions. The redistribution of the charges creates electric dipoles in the SWNTs. The interactive force between the dipoles and the non-uniform electric field is called dielectrophoretic force *F*, which is expressed as [26,27]:

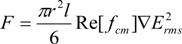
(1)
where the term *πr^2^l/6* is a geometry factor that contains the volume information of the nanotube, *e_m_* is the permittivity of the solvent medium, *Re*[*f_cm_*] is the real number part of the Clausius-Mossotti factor *f_cm_*, and *▽E_rms_* is the gradient of the root mean square of the external electric field. As a result of the dielectrophoretic force, the SWNTs are able to move and rotate in the solution to follow the directions of the electric field lines.

**Figure 4:**
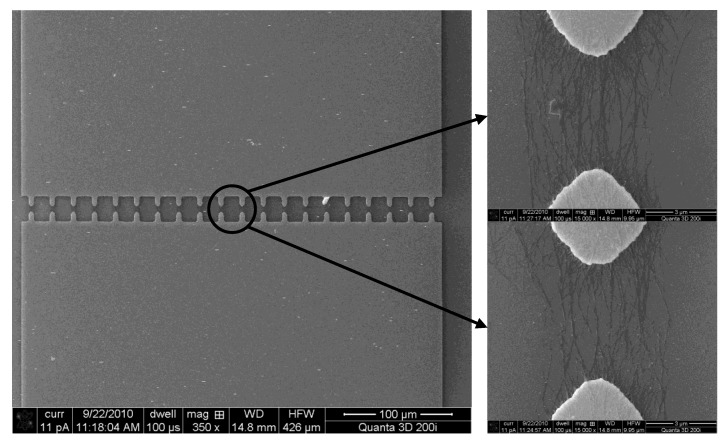
SEM images of the sensor structure and the dielectrophoretically aligned SWNTs.

To align the SWNTs, a small drop of the SWNT solution is placed on the substrate to cover the electrodes using a syringe. Next, the function generator is switched on; an AC electric field is generated between the electrodes. The electric field exerts dielectrophoretic forces on the SWNTs and forces them to rotate along the field lines. As a result, the SWNTs are rapidly attracted by the dielectrophoretic force and landed on the substrate across the electrodes. An instant voltage drop of 1–2 V can be observed from the oscilloscope during the capturing event. The typical time range of the dielectrophoresis process in our investigation is 30 s. Next, the solution residual is removed with a syringe. The dielectrophoretically assembled SWNTs are inspected with an SEM, as shown in Figure 4. Although the exact number of aligned SWNTs varies from one electrode pair to another, the alignment results and the amount of aligned SWNTs are relatively consistent. The SWNTs are mainly in the form of bundles and they can only be observed in between the electrode pairs, where the electric field has the highest magnitude.

### 3.2. pH Sensitivity

Early study showed that the pristine SWNT is a p-type material, *i.e.*, the majority charge carriers are holes. The acid-functionalized SWNTs also demonstrated p-type characteristics when used in electronic devices [28]. When such SWNTs are submerged in pH buffer solutions, hydrogen (H^+^) and hydroxide (OH^−^) ions can interact with the carboxyl groups covalently attached to the SWNTs. Hydrogen (H^+^) as a proton is able to bind to the surface of the SWNT and accept one electron from the p-orbital of the nanotube. This induces the generation of a positive hole in the SWNT and increases its conductance [11]. At the same time, the hydroxide (OH^−^) ions cause the opposite effects, decreasing the conductance of the SWNT. Consequently, the concentrations of H^+^ and OH^−^ ions affect the generation of holes and electrons in the SWNTs. The conductance of the SWNTs is therefore changed by this protonation/deprotonation process [17].

Because pH sensitivity measurement is conducted in an aqueous environment, a control experiment is needed to ensure that the majority of the charges transmit through the SWNTs instead of the solutions. In this experiment, the electrical conductivities of all the aqueous media—the pH buffer solutions and the DI water—are obtained by placing these solutions on open electrodes without the SWNTs. The semiconductor analyzer is used to measure the resistances of these solutions. The results show that the resistances of the solutions are almost infinite. This means that the solutions have much lower conductivities than the SWNTs. Therefore, for the SWNT-based pH sensors, the current is transmitted through the SWNTs almost exclusively. Any change recorded by the analyzer is caused by the resistance change of the SWNTs.

**Figure 5:**
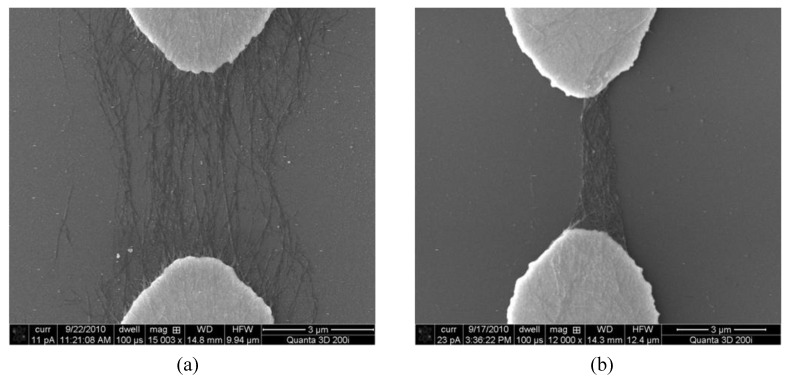
**(a)** An SEM image of sparsely distributed SWNTs across the electrodes right after the dielectrophoresis deposition. **(b)** An SEM image of congregated SWNTs after the droplet placing and removing steps.

In our investigation, it is observed that the droplet placing and removing steps need to be performed a few times (10–15) before the sensor shows a stable response to pH solutions. We believe that during the first few instances of sensor wetting and drying, some SWNTs lose the connection with the electrodes and are washed away in the process. Therefore, only the initially strongly-bond SWNTs remain on the surface. The effect can be demonstrated by monitoring the SWNTs before and after the wetting and drying steps. Figure 5(a) shows the aligned SWNTs right after the dielectrophoresis process but before the pH sensing steps. The SWNTs are sparsely distributed and loosely connected to the electrodes. In comparison, Figure 5(b) shows the remaining SWNTs after a few instances of droplet placing and removing. The originally sparsely distributed SWNTs are now tightly congregated together and form a dense network. This is attributed to liquid surface tension when the solution residual evaporates. The surface tension pulls the SWNTs together and reinforces the connection between the SWNTs and the electrodes. In addition, the loosely-bond SWNTs are removed during the process. As a result, the sensor becomes more stable and demonstrates higher repeatability after these droplet placing and removing steps.

**Figure 6:**
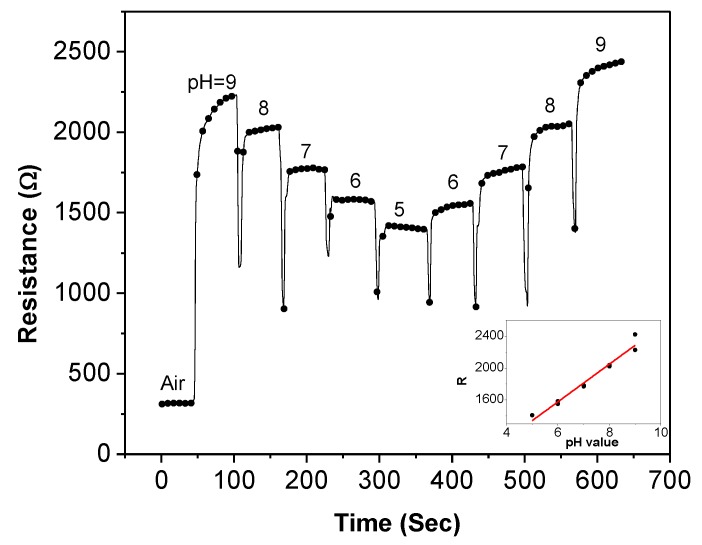
The recorded resistance history of a SWNT-based sensor when exposed to pH buffers from pH-9 to pH-5 and then to pH buffers from pH-5 to pH-9.

After the SWNTs become stabilized, the sensor is exposed to buffer solutions for pH sensitivity measurement. For each solution, a syringe is used to place a droplet on top of the SWNTs. During the measurement, the sensor is first exposed to the five solutions in the sequence of pH-9 to pH-5, and then it is exposed to the same solutions in the opposite sequence of pH-5 to pH-9. Figure 6 illustrates the recorded resistance history (resistance-time plot) of the sensor during the measurement. The real-time resistance change at different pH values proves that the sensor is highly sensitive to the pH variation. When the sensor is exposed to air, the resistance is approximately 316 Ω. After the pH-9 buffer is placed on the sensor, the resistance is rapidly increased to 2,226 Ω. The pH-9 buffer droplet is then removed after approximately 50 s when the sensor resistance is relatively stable. Next, the pH-8 buffer is dropped on the sensor immediately after the pH-9 buffer removal. The average sensor resistance is decreased by 206 Ω. The same step is repeated for pH-7, pH-6, and pH-5 solutions. Similar amount of decrement of sensor resistance can be observed for each step. On the other hand, almost equal amount of increment of sensor resistance is observed using the solutions with increasing pH values. Based on the recorded data, the sensor resistance and the pH values have a linear relationship of *R* (Ω) = 236.3 × pH + 159, as shown in the inset of Figure 6. This equation indicates that the absolute sensitivity of this particular sensor is 236.3 Ω/pH. However, we want to point out that even though the sensors are fabricated under the same condition, it is still challenging to deposit the exact same amount of SWNTs over the electrodes on every sensor. Consequently, the SWNT-based thin-film sensors often suffer from performance variation with absolute values vary from device to device. Another possible reason for the performance variation is from the material itself—SWNTs contain both metallic and semiconducting nanotubes. The uncertainty of the film composition causes the property variation of the deposited SWNTs. Therefore, a more consistent method needs to be used. In our investigation, the sensing repeatability and the normalized resistance of various sensors are used to characterize the performance of the SWNT-based sensors.

### 3.3. Sensing Repeatability

The sensing repeatability of the SWNT-based sensors is estimated through the measurement of five different sensors following the same procedure described above. All sensors demonstrate similar behaviors with resistance-time plots similar to the one shown in Figure 6. The average resistance at each pH value is then calculated for each sensor. The normalized resistance is adopted to evaluate the sensor performance; it is defined as:

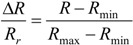
(2)
where *ΔR/R_r_* is the normalized sensor resistance, *ΔR* is sensor resistance relative to the lowest sensor resistance, *R_r_* is the range of sensor resistance in the pH sensing test, *R* is the absolute value of sensor resistance, *R_max_* is the highest sensor resistance, and *R_min_* is the lowest sensor resistance.

**Figure 7:**
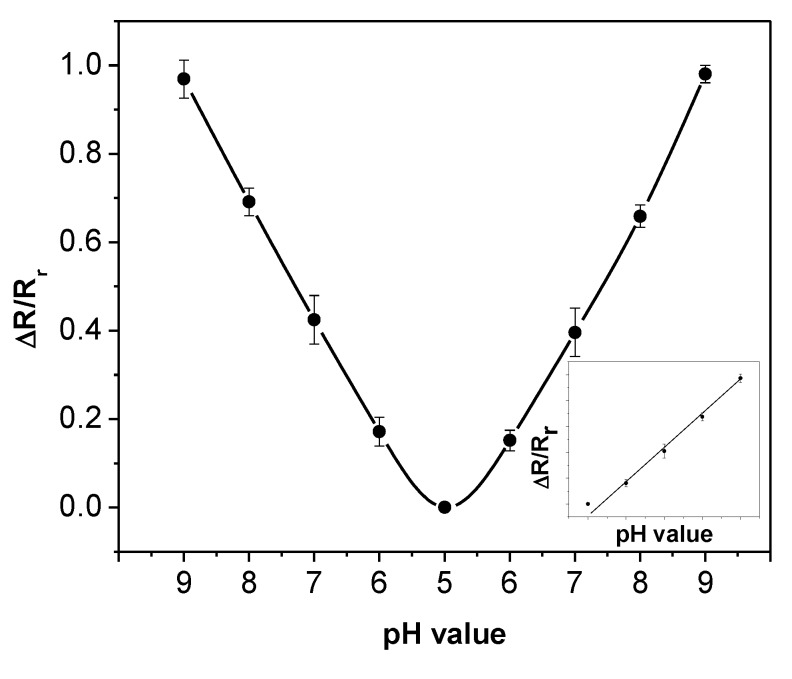
Normalized resistances *versus* pH values for five SWNT-based sensors. Inset: the linear relationship between the normalized sensor resistance and the pH value.

Figure 7 shows the average values (dots) and standard deviations (error bars) of the normalized resistances at different pH values for the five sensors. All the sensors follow the same trend of pH sensitivity and the values vary in a small range. This indicates that the pH sensitivity is highly repeatable for multiple sensors. As the pH value decreases from 9 to 5, the normalized sensor resistance drops proportionally. The opposite trend can be seen from the right half of the figure where the pH value increases. The figure also demonstrates that the sensor resistance depends solely on the pH value rather than the testing order of the pH solutions. The measurement data from the entire process are summarized and re-plotted as the inset of Figure 7, which shows a linear relationship between the normalized sensor resistance and the pH values in the range of 5 to 9. The linear relationship can be described as:

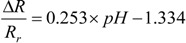
(3)
This equation provides the calibration standard of the SWNT-based sensors using pH-5 to pH-9 buffer solutions.

### 3.4. Response Time

**Figure 8:**
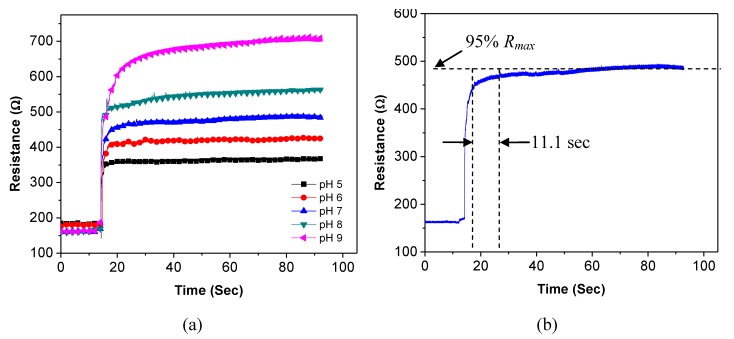
**(a)** pH response under buffer solutions from pH-5 to pH-9. **(b)** The response time of the sensor under the pH-7 buffer solution.

Response time represents how long it takes for the sensor to reach a steady state. To obtain accurate results, the response time of the sensor is investigated with an increased sampling rate of 100 points per sec. The initial resistance of the sensor in air is recorded by the analyzer. Next, a droplet of the pH buffer is placed on the sensor. After the resistance of the sensor reaches a stable value, the pH buffer is removed with the syringe. Next, the sensor is rinsed with DI water to remove the buffer residual. The same experiment is performed for all the pH buffer solutions and the results are illustrated in Figure 8(a). All curves show instant increases after the buffer solutions cover the sensor. The pH-5 buffer yields the minimum sensor resistance. As the pH value increases, the resistance of the sensor increases. In addition, the measurement results show that the response time of the sensor is not a constant; instead, it depends on the pH value. In our investigation, the response time of the sensor is defined as the time for the resistance to reach 95% of its maximum value. An example curve is illustrated in Figure 8(b) for the pH-7 buffer. It takes the sensor 11.1 s for its resistance to reach 95% of the maximum value of 495 Ω.

Using the same method, the sensor response times at different pH values are calculated and summarized in Table 1. The sensor response time increases when the pH value increases. The pH-dependent response time may be caused by the interaction speed between the hydrogen ions and the SWNTs. As the concentration of hydrogen ions becomes lower, the protonation/deprotonation process becomes slower. Consequently, it takes longer time for the SWNTs to reach a stable resistance.

**Table 1:** Sensor response time under different pH values.

pH value	5	6	7	8	9
Response time (s)	2.26	3.08	11.1	17.05	23.82

### 3.5. Long-Term Stability

**Figure 9:**
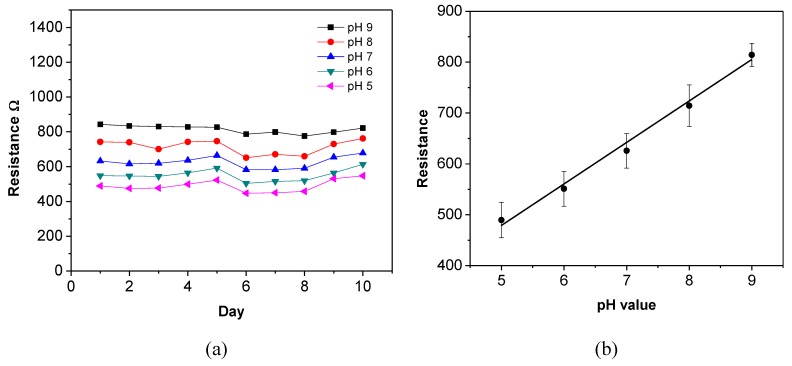
Resistance variation of one sensor in five pH buffer solutions in 10 days.

Long-term stability is an important parameter for sensors, especially for pH sensors because they are used in aqueous environments which usually contain many affecting factors. Ideally, the sensors should be able to maintain their sensitivity for a long period of time. To study the long-term stability, a SWNT-based sensor is characterized every 24 h for 10 consecutive days under the same experimental conditions. Figure 9(a) shows the recorded absolute resistances of the sensor in different pH buffer solutions during this period. The values are relatively stable. The sensor remains sensitive to the pH buffer solutions after 10 days; and its sensitivity is relatively consistent during this period. To estimate the relationship between the absolute resistance and the pH value, the obtained data are re-plotted in Figure 9(b). The dots represent the average values and the error bars represent the standard deviations of the sensor resistance. The resistance-pH plot verifies that the sensor maintains its linear relationship between the resistance and the pH values during the 10-day period. 

## 4. Conclusions

In conclusion, we have developed and characterized high-performance pH sensors using dielectrophoresis aligned SWNTs and “teeth”-like electrodes. The fabrication techniques including photolithograph, wet etching, and dielectrophoresis, are simple and inexpensive. Because the process is compatible with industry standard microfabrication technology, the sensor fabrication procedure has high potential to be extended to large-scale device production. Furthermore, we have demonstrated that the electrodes and the aligned SWNTs can be used as pH sensors. The resistance of the SWNTs is highly dependent on the pH value. A linear relationship between the normalized resistance and the pH value in the range of 5–9 is observed. The sensor performance is highly repeatable; the characterization shows similar results for five sensors. The normalized resistance-pH plot from the five sensors is highly linear with small variation. The sensors demonstrate fast response, providing high potential for real-time sensing applications. In addition, they are able to maintain the same sensitivity for a long period of time after repeated measurements. Based on our investigation, we believe that the SWNT-based sensors can be developed and applied in a wide range of areas. Although SWNT-based sensor research is still in its early stages and needs further investigation, we believe that with more research groups and efforts involved, the performance of these sensors can be dramatically increased in the near future.
